# Functional characterization of two CITED3 homologs (gcCITED3a and gcCITED3b) in the hypoxia-tolerant grass carp, *Ctenopharyngodon idellus*

**DOI:** 10.1186/1471-2199-10-101

**Published:** 2009-11-03

**Authors:** Patrick KS Ng, Sung-Kay Chiu, Theresa FN Kwong, Richard MK Yu, Minnie ML Wong, Richard YC Kong

**Affiliations:** 1 Department of Biology and Chemistry, City University of Hong Kong, Kowloon Tong, Hong Kong Special Administrative Region, PR China; 2 Department of Biochemistry, Chinese University of Hong Kong, New Territories, Hong Kong Special Administrative Region, PR China; 3 School of Environmental and Life Sciences, University of Newcastle, New South Wales, Australia

## Abstract

**Background:**

CITED proteins belong to a family of non-DNA-binding transcriptional co-regulators that are characterized by a conserved ED-rich domain at the C-terminus. This family of genes is involved in the regulation of a variety of transcriptional responses through interactions with the CBP/p300 integrators and various transcription factors. In fish, very little is known about the expression and functions of CITEDs.

**Results:**

We have characterized two closely related but distinct CITED3 genes, *gcCited3a *and *gcCited3b*, from the hypoxia-tolerant grass carp. The deduced gcCITED3a and gcCITED3b proteins share 72% amino acid identity, and are highly similar to the CITED3 proteins of both chicken and *Xenopus*. Northern blot analysis indicates that the mRNA expression of *gcCited3a *and *gcCited3b *is strongly induced by hypoxia in the kidney and liver, respectively. Luciferase reporter assays demonstrated that both gene promoters are activated by gcHIF-1. Further, ChIP assays comparing normal and hypoxic conditions reveal differential *in vivo *binding of gcHIF-1 to both gene promoters in kidney and liver tissues. HRE-luciferase reporter assays demonstrated that both gcCITED3a and gcCITED3b proteins inhibit gcHIF-1 transcriptional activity, and GST pull-down assays confirmed that both proteins bind specifically to the CH1 domain of the grass carp p300 protein.

**Conclusion:**

The grass carp *gcCITED3a *and *gcCITED3b *genes are differentially expressed and regulated in different fish organs in response to hypoxic stress. This is the first report demonstrating *in vivo *regulation of two closely-related CITED3 isogenes by HIF-1, as well as CITED3 regulation of HIF-1 transcriptional activity in fish. Overall, our findings suggest that unique molecular mechanisms operate through these two gcCITED3 isoforms that likely play an important regulatory role in the hypoxic response in the grass carp.

## Background

Cells and tissues respond to low oxygen levels by stabilizing the HIF-1 transcription factor, which controls the expression of over 100 different genes that are involved in adaptation and survival [1]. These include genes involved in erythropoiesis (e.g. *EPO*), vasculogenesis (e.g. *VEGF*), glucose metabolism (e.g. *GLUT1 *and *GLUT4*), and fibrogenesis. HIF-1 is a heterodimeric DNA-binding protein composed of an oxygen-sensitive HIF-1α subunit and a constitutively expressed HIF-1β subunit (also known as the aryl hydrocarbon receptor nuclear translocator, or ARNT) [2]. In the presence of oxygen, HIF-1α is hydroxylated by a prolyl hydroxylase [3]. This triggers its interaction with the pVHL protein, which targets HIF-1α for degradation by the 26S proteosome [4]. In the absence of oxygen, prolyl hydroxylase activity is inhibited. This results in the stabilization of HIF-1α and the subsequent translocation of the HIF-1α subunit into the nucleus where it binds HIF-1β and forms transcriptionally active HIF-1. HIF-1 regulates gene expression by interacting with sequence-specific hypoxia-responsive elements (HREs) found in either the 5'-flanking, 3'-flanking, or intronic regions of HIF-responsive genes. The HRE was first identified as a 256-bp sequence in the 3'-flanking region of the human *EPO *gene [5].

The CITED [cAMP-responsive element-binding protein (CBP)/p300-interacting transactivator with glutamic acid/aspartic acid-rich tail] proteins belong to a family of transcriptional cofactors that is characterized by a conserved ED-rich domain at the C-terminus. The biological properties of CITED proteins include modulating a variety of cellular and developmental processes [6,7] and responding to diverse biological [8] and environmental stimuli [9,10]. To date, four different CITED homologs have been reported in vertebrates. CITED2, which can function as an activator and a repressor depending on the tissue, is the most extensively studied of the four. In the initial description, CITED2 was shown to function as a repressor of hypoxia-inducible factor-1 (HIF-1) through competition for binding to the CH1 domain of CBP/p300 [11]. The LPXL (Leu-Pro-X-Leu) motifs in both CITED2 and HIF-1α interact with overlapping binding sites on the CH1 domain of p300 [12]. CITED2 has been reported to bind this same region with 33-fold greater affinity than HIF-1α [9]. Genetic evidence indicates that loss of CITED2 is associated with increased activation of HIF-1 target genes [13], supporting the hypothesis that CITED2 is a negative regulator of HIF-1α. Conversely, CITED2 functions as a co-activator for several transcription factors, such as AP-2 [14], PPAR-α, and PPAR-γ [15], by linking them to CBP/p300. Cellular responses to TGF-β are largely mediated by the Smad proteins, which serve as both transcription factors and transcriptional co-regulators. CITED2 is an important regulator of TGF-β signaling through direct association with Smad2 and Smad3 [16]. Members of the CITED protein family may also play an important role in the regulation of reproductive functions. Studies have shown that CITED2 interacts with the LIM domain of the Lhx2 transcription factor to enhance transcription of the glycoprotein α-subunit gene [17]. Furthermore, CITED1 has been shown to bind to the estrogen receptor ER-α and enhance the transcription of estrogen-inducible genes such as TGF-α [7].

CITED3 is the least studied member of the CITED family. Previous studies have shown that it is highly expressed during the early stages of embryonic development in the mesonephric tubules and eye in the chicken [18], the pronephros and eye in the frog [19], and the kidney of adult grass carp [20]. While much is known about the distribution and regulation of the CITED proteins in mammals, very little is known about these proteins in fish. Fish are ideal models to study molecular and cellular adaptation to hypoxia, as fluctuations in environmental oxygen availability have played an important role in the evolution of these animals [21]. The expression patterns and functions of the CITED proteins remain largely unknown in cyprinids. Our group has previously reported the presence of a hypoxia-responsive CITED3 cDNA in grass carp [20]. In an attempt to gain broader insights into the evolution and possible functions of the different CITED genes in fish, this study describes our findings on two closely-related but distinct CITED3 homologs - gcCITED3a and gcCITED3b - from the hypoxia-tolerant grass carp. We present evidence for a differential role of these two *gcCITED3 *genes for adaptation to hypoxia in grass carp based on their *in vivo *mRNA expression and response pattern to short- and long-term hypoxia, as well as *in vitro *gene transactivation studies.

## Results

### Identification of two CITED3 genes in grass carp

**Figure 1:**
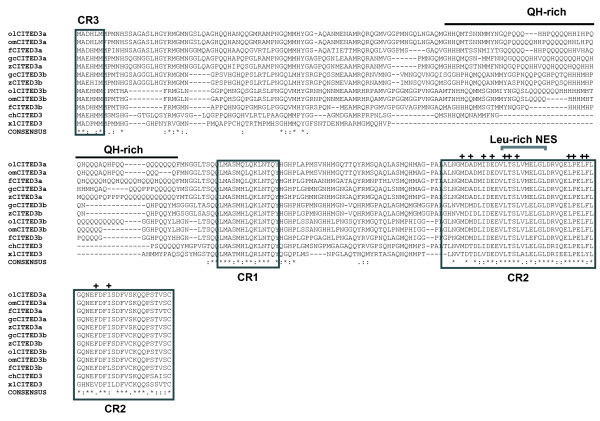
**Amino acid sequence alignment of gcCITED3a and gcCITED3b with related homologs from other fish species**. Amino acid residues in the CR1, CR2 and CR3 conserved domains typically found in CITED proteins are boxed and labeled. Amino acid residues in the CR2 domain that interact with the CH1 domain of CBP/p300 are indicated with a plus (+). The QH-rich domain of fish CITED3a/CITED3b is highlighted by a grey overline. The leucine-rich nuclear export signal (Leu-rich NES) is indicated above the alignment with a parenthesis. olCITED3a and olCITED3b, *Oryzias latipes *CITED3a and CITED3b; omCITED3a and omCITED3b, *Oryzias melastigma *CITED3a and CITED3b; fCITED3a and fCITED3b, Fugu CITED3a and CITED3b; gcCITED3a and gcCITED3b, grass carp CITED3a and CITED3b; zCITED3a and zCITED3b, zebrafish CITED3a and CITED3b; xlCITED3, *Xenopus *CITED3; chCITED, chicken CITED3.

We have previously described a full-length CITED3 cDNA from grass carp that shows high similarity to chicken and *Xenopus *CITED3 [20]. In this study, degenerate primers, targeting consensus sequences derived from a multiple alignment of the CITED3 open reading frames (ORFs) of grass carp [GenBank: AY225852], zebrafish [GenBank: AF359242], chicken [GenBank: AF261079] and *Xenopus *[GenBank: AI031460], were used for RT-PCR on total RNA extracted from the kidney of a grass carp that was exposed to hypoxia for 4 h. A distinct cDNA fragment, cix1 (0.7 kb), with a partial ORF that shared high sequence identity with the grass carp CITED3 ORF was identified. Using 5'- and 3'-RACE PCR, a 3032-bp full-length cDNA, CIX1, was obtained. DNA sequencing showed that CIX1 contains 5'- and 3'-untranslated (UT) regions of 122 bp and 2187 bp, respectively, and an ORF (723 bp) that specifies a protein of 240 amino acids with a predicted molecular weight of 26.3 kDa. In agreement with this finding, a single mRNA transcript of approximately 3.0 kb was detected by Northern blot (data not shown). A pairwise sequence comparison demonstrated that CIX1 shares particularly high similarity with the CITED3 proteins of grass carp (72%); zebrafish (69.4%); and chicken (64.1%), but only moderate similarity (42 - 49%) with the CITED2 protein, and low similarity (<42%) with the CITED1 and CITED4 proteins from different vertebrate species. Moreover, the primary structure of the CIX1 and grass carp gcCITED3 [GenBank: AAO48505] proteins contain the characteristic CR1, CR2, and CR3 domains that are highly similar to the homologous domains present in the chicken and *Xenopus *CITED3 proteins (Figure 1). Taken together, these data strongly suggest that CIX1 is a novel CITED3-like homolog. We refer to this gene as *gcCited3b*, and the previously described gcCITED3 as *gcCited3a *[20]. This designation was chosen since the primary sequences and structures of these two homologs are most similar to known CITED3 proteins. The nucleotide sequence data of *gcCited3a *and *gcCited3b *have been deposited in the GenBank database as GenBank: AY225852 and GenBank: EU450668, respectively.

### Characteristics of the deduced gcCITED3a and gcCITED3b proteins

Sequence alignment of the grass carp gcCITED3a and gcCITED3b proteins with homologs from pufferfish (*Tetraodon nigrovindis*), zebrafish (*Danio rerio*), Japanese medaka (*Oryzias latipes*), marine medaka (*Oryzias melastigma*) and Fugu (*Fugu rubripes*) indicated extensive sequence similarity in the CR1, CR2, and CR3 domains (Figure 1). CR2 is the characteristic domain of the CITED protein family and is present in all known CITED proteins. Notably, a particularly high degree of sequence conservation is observed in the core CR2 motif, which is known to interact with the CH1 domain of the p300/CBP co-transactivator [12]. Importantly, a glutamine and histidine-rich (QH-rich) region (amino acid positions 95 - 131 in gcCITED3a) is found only in the CITED3a and CITED3b homologs in fish but is absent in the chicken and *Xenopus *CITED3 (Figure 1), and mammalian CITED1, CITED2, and CITED4 proteins (data not shown). In addition, a leucine-rich nuclear-export signal (LMSLVVELGL), which presumably contributes to the sub-cellular localization of the CITED proteins [22], is also conserved within the CR2 domain of all the fish CITED3a and CITED3b proteins.

### Phylogeny of fish CITED3a and CITED3b proteins

**Figure 2:**
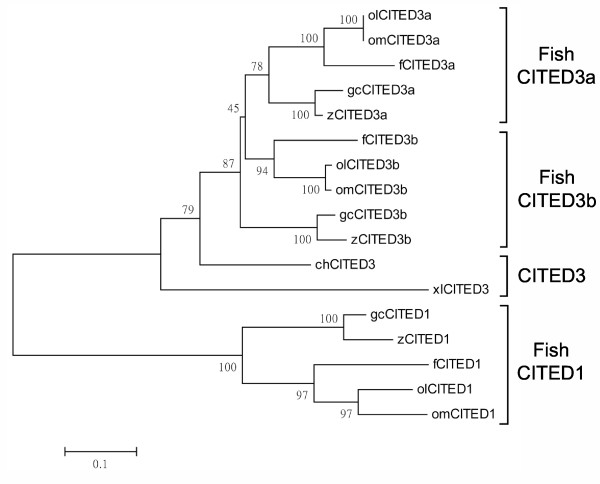
**Phylogeny of fish gcCITED3a and gcCITED3b proteins**. The tree was constructed by the neighbor-joining method of the MEGA v3.1 program using fish CITED1 proteins as outgroup. The bootstrap support for each branch (1000 replications) is shown. The branch lengths are proportional to the number of substitutions between sequences. The GenBank/EMBL/Swissprot accession numbers of the CITED sequences used are given in Table 1.

*In silico *screens of the zebrafish, Japanese medaka, *Fugu *genome and/or EST and NCBI GenBank databases identified a number of fish homologs that share substantial sequence similarity with the gcCITED3a and gcCITED3b proteins. Similar screens of various mammalian databases with CITED3, CITED3a, or CITED3b failed to identify any CITED3-like sequences, suggesting that these genes are absent in mammals. The phylogenetic relationship between different CITED3 proteins was analyzed by the neighbor-joining method using fish CITED1 proteins as the outgroup (see Table 1). As shown in Figure 2, CITED3 proteins are separated with high bootstrap support into three different sub-clades: fish-specific CITED3a and CITED3b, and avian/*Xenopus *CITED3.

**Table 1:** Deduced CITED1 and CITED3 proteins for phylogenetic analysis.

Abbreviations	Standings	Accession numbers
olCITED3a	*Oryzias latipes *CITED3a	scaffold 164^#^
omCITED3a	*Oryzias melastigma *CITED3a	[GenBank: ACA52075]
fCITED3a	*Fugu *CITED3a	scaffold 2627*
gcCITED3a	grass carp CITED3a	[GenBank: AAO48505]
zCITED3a	zebrafish CITED3a	[GenBank: AAK43715]
fCITED3b	*Fugu *CITED3b	scaffold 301*
olCITED3b	*O. latipes *CITED3b	scaffold 8^#^
omCITED3b	*O. melastigma *CITED3b	[GenBank: ACA52076]
gcCITED3b	grass carp CITED3b	[GenBank: ACA48500]
zCITED3b	zebrafish CITED3b	[GenBank: XP_695901]
chCITED3	chicken CITED3	[GenBank: AAF76148]
xlCITED3	*Xenopus *CITED3	[GenBank: BC106415]
gcCITED1	grass carp CITED1	[GenBank: ACA48501]
zCITED1	zebrafish CITED1	[GenBank: ACA02700]
fCITED1	*Fugu *CITED1	[GenBank: CAF90007]
olCITED1	*O. latipes *CITED1	scaffold 204^#^
omCITED1	*O. melastigma *CITED1	[GenBank: ACA52074]

^# ^CITED proteins deduced from the Japanese medaka genome database

* CITED proteins deduced from the Fugu genome database (JGI *Fugu *genome v3.0)

The abbreviations are listed from top to bottom in accordance with Figure 2.

### In vivo mRNA expression and response patterns of gcCITED3a and gcCITED3b to short- and long-term hypoxia

**Figure 3:**
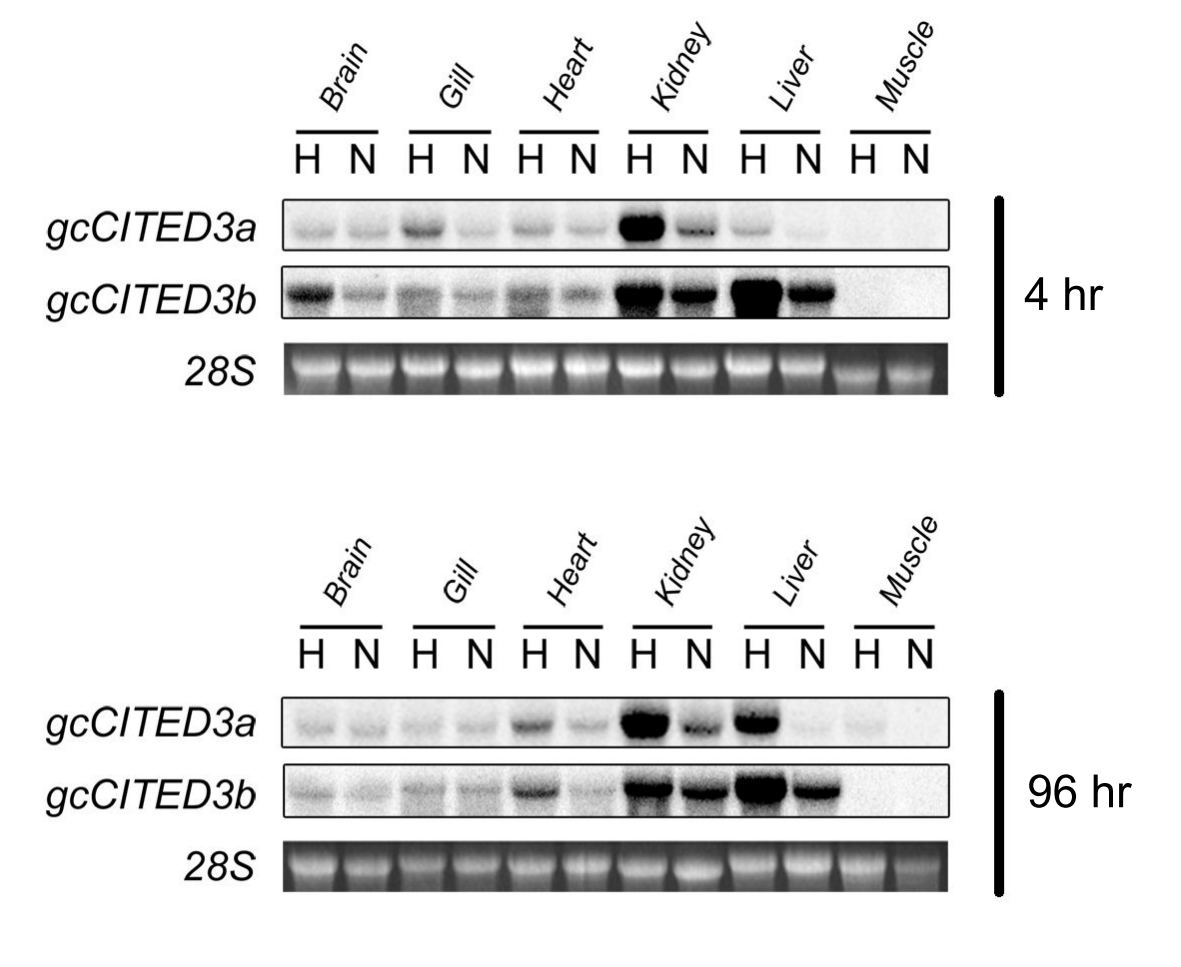
**Northern blot analysis of *gcCited3a *and *gcCited3b***. Fish tissues (brain, gill, heart, kidney, liver, and muscle) were isolated from normoxic (N) and hypoxic (H) grass carp following 4 h and 96 h of exposure as indicated. Total RNA from each tissue was isolated and analyzed by Northern hybridization using gene-specific probes for *gcCITED3a *and *gcCITED3b*. Ethidium bromide-stained 28S rRNA is shown as the loading control. A representative Northern blot derived from the tissues of one normoxic and one hypoxic fish (from a total of three in each group) is shown here.

To examine the *in vivo *expression pattern of *gcCited3a *and *gcCited3b *in response to hypoxia, grass carp (*n *= 3) were exposed to normoxia (7.0 mg O_2_/l) and hypoxia (0.5 mg O_2_/l) for 4 h and 96 h. Total RNA was isolated from six different tissues (brain, gill, heart, kidney, liver, and muscle) of each fish at each time point for Northern blot analysis. Overall, the normoxic mRNA expression and hypoxic induction pattern of the *gcCited3a *and *gcCited3b *genes in different grass carp tissues were consistent between all three replicate blots, and a representative autoradiogram is shown in Figure 3. Under normoxic conditions, *gcCited3a *(1.6-kb mRNA) expression was detected in all tissues examined except muscle. After 4 h of hypoxia, a significant induction of *gcCited3a *was observed in the kidney, and a mild induction was identified in the gill and liver. The brain, heart, and muscle tissues showed no change in the expression of *gcCited3a*. After 96 h of hypoxia, a marked induction of expression was observed in the kidney and liver, a modest induction was detected in the heart, and no change in *gcCited3a *expression was observed in the brain, gill, or muscle.

Under normoxic conditions, *gcCited3b *mRNA expression was also detected in all tissues examined except the muscle, and the highest level was detected in the kidney and liver. After 4 h of hypoxia, *gcCited3b *mRNA expression was induced in all tissues except the muscle. The highest induction was observed in the kidney and liver. After 96 h of hypoxia, the induction of *gcCited3b *expression was detected in the brain, gill, heart, and kidney.

In all replicates of each hypoxic tissue, the mRNA expression level of *gcCited3a *and *gcCited3b *was normalized against the 28S rRNA and compared to their normoxic counterparts. If the expression level following hypoxia was equal to the normoxia control group, the hypoxia:normoxia expression ratio should be equal to one. In contrast, this ratio is expected to be significantly greater than one in the case of hypoxic induction of the genes. The 4 h and 96 h datasets for each tissue were combined and a non-parametric χ^2 ^test was performed to examine the significance of the induction. The analysis indicated that the expression of both *gcCited3a *and *gcCited3b *was significantly different in the kidney and liver following hypoxia compared to the normoxic controls (*p *< 0.05).

### Activation of gcCited3a and gcCited3b promoter activity by gcHIF-1 in CHO cells

**Figure 4:**
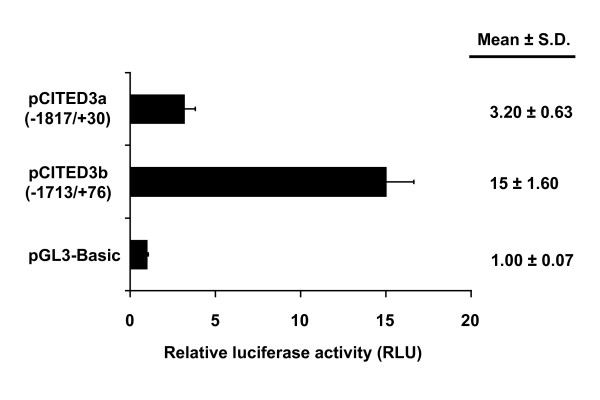
**Transcriptional activation of *gcCited3a *and *gcCited3b *gene promoters and identification of gcHIF-1 binding sites by ChIP-PCR**. CHO cells were transiently transfected with the pCITED3a(-1817/+30), pCITED3b(-1713/+76) or pGL3-Basic luciferase vectors together with pCMV-gcHIF1α, pCMV-gcARNT2b (HIF-1 complex) and pSVβ-gal plasmid or the empty pCMV vector. Luciferase was normalized against β-gal activity and data represent the means ± S.D. of three independent experiments. Asterisks indicate significant differences, *P *≤ 0.05, between pCITED3a and pCITED3b promoter constructs and the pGL3-Basic vector.

**Figure 5:**
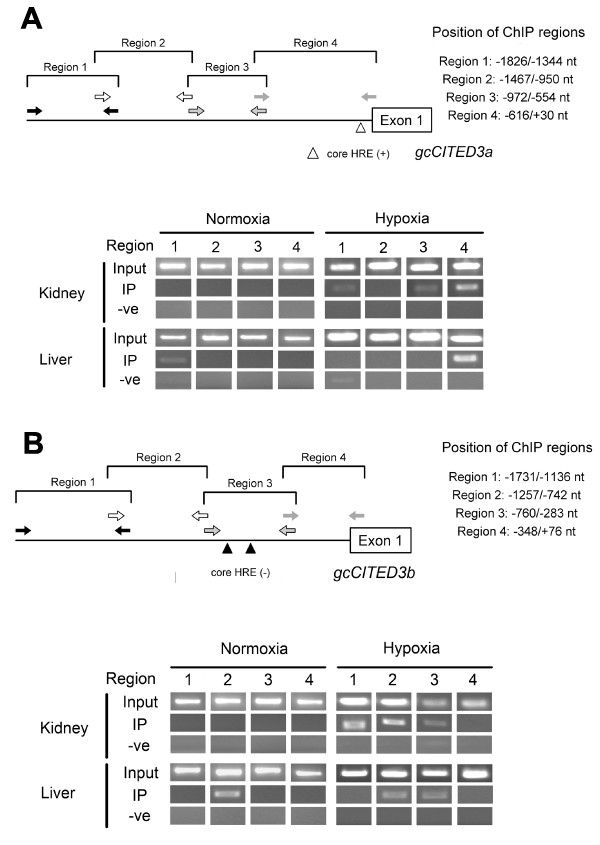
**ChIP-PCR analysis of gcHIF-1 binding to the *gcCited3a *and *gcCited3b *gene promoters**. *A*. The primer-sets (indicated by opposing arrows of the same grey scale) for ChIP-PCR amplification of different regions of the 5'-flanking sequence of *gcCITED3a *are shown, and the regions analyzed are demarcated on the right panel. *B*. The primer-sets (indicated by opposing arrows of the same grey scale) for ChIP-PCR amplification of different regions of the 5'-flanking sequence of *gcCITED3b *are shown, and the regions analyzed are demarcated on the right panel. Representative ChIP results from kidney (upper panel) and liver (lower panel) of normoxic (left panel) and hypoxic (right panel) fish. The PCR products of samples without immunoprecipitation (Input), immunoprecipitated with anti-gcHIF-1α anti-serum (IP) and water (-ve) were resolved by gel electrophoresis. A PCR band appears if a DNA region is bound by gcHIF-1 in the IP (immunoprecipitated) samples.

To investigate whether gcHIF-1 regulates the *gcCited3a *and *gcCited3b *gene promoters, Chinese hamster ovary (CHO) cells were transiently transfected with the pCITED3a(-1817/+30) or pCITED3b(-1713/+76) luciferase construct, together with pBK-CMV-gcHIF1α [23], or the empty pBK-CMV expression vector. As shown in Figure 4, luciferase activity was strongly activated in CHO cells co-transfected with pCITED3a(-1826/+30) and the pBK-CMV-gcHIF-1α (ca. 3.2-fold), while cells similarly transfected with pCITED3b(-1731/+76) exhibited an even greater HIF-1-induction (ca. 15-fold), when compared to control cells transfected with the empty pGL3-Basic vector (*p *< 0.05). Interestingly, promoter sequence analysis revealed the presence of a single core HRE (CGTG) at position -59/-56 of the 5'-flanking region of the *gcCited3a *gene (Figure 5A), while two putative HREs in the reverse DNA strand (CACG) were detected at -629/-626 and -510/-507 of the *gcCited3b *gene (Figure 5B).

At the time of the study, no functional HRE had been reported in fish, and the binding of the HIF-1 transcription factor to consensus and non-consensus HREs had only been speculated [24]. In order to determine whether the gcHIF1 binds to the *gcCited3a *and *gcCited3b *promoters *in vivo*, ChIP assays were performed on grass carp kidney and liver tissues using PCR primer sets that spanned four overlapping regions (ca. 500 bp each) within the 5'-flanking sequences of the *gcCited3a *and *gcCited3b *genes. Under normoxic conditions, gcHIF-1 did not bind to the *gcCited3a *promoter; however gcHIF-1 binding was detected in region 4 (nucleotide position -616/+30) of the *gcCited3a *promoter (which contains a single core HRE at -59/-56) in kidney and liver tissues of fish exposed to 4 h of hypoxia (Figure 5A). These data demonstrate that gcHIF-1 formed a specific complex at the *gcCited3a *promoter *in vivo*. In contrast, three different gcHIF-1 binding patterns were observed at the *gcCited3b *promoter following ChIP: specific gcHIF-1 binding was detected at nucleotide positions -1731/-1136 (region 1) and -1257/-742 (region 2), neither of which contain a consensus or core HRE sequence, in the kidney of hypoxic fish, but not in normoxic kidney (Figure 5B). Additionally, gcHIF-1 binding was detected in region 2 of *gcCited3b *in the liver of both normoxic and hypoxic fish. Finally, gcHIF-1 binding was specifically detected at nucleotide position -760/-283 (region 3, which contains two core HRE motifs in the reverse orientation) in the liver of hypoxic fish.

### Inhibition of gcHIF-1 transactivation of HRE-luciferase activity by gcCITED3a and gcCITED3b

**Figure 6:**
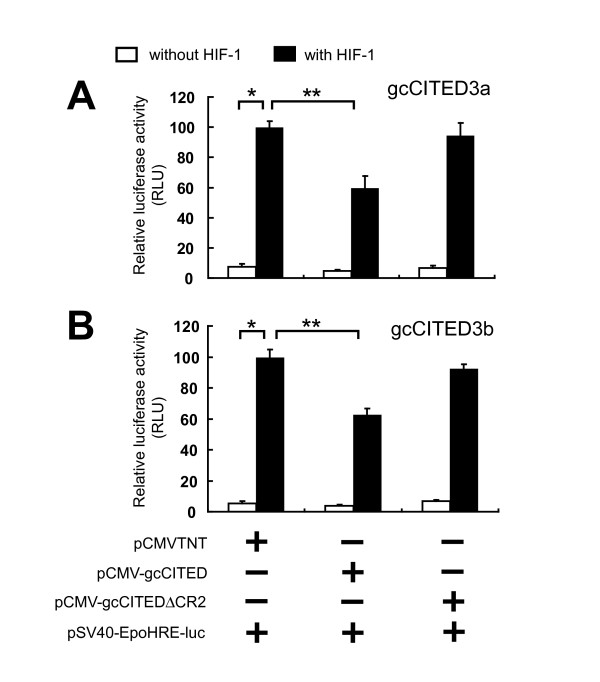
**Transactivation of HRE-luciferase activity by gcHIF-1 is inhibited by gcCITED3a and gcCITED3b**. CHO cells were co-transfected with a pSV40-EpoHRE-luciferase reporter and pSV-β-gal vector (control for transfection efficiency) along with pBK-CMV-gcHIF-1α and pBK-CMV-gcARNT2b or empty pCMV-TNT vector. Wild-type pCMV-gcCITED3a or deletion mutant plasmid pCMV-gcCITED3aΔCR2 (panel A); or pCMV-gcCITED3b or deletion mutant plasmid pCMV-gcCITED3bΔCR2 (panel B) was also co-transfected with pBK-CMV-gcHIF-1α. Relative luciferase activity is the ratio of luciferase over β-galactosidase activity. Transfections were performed in triplicates and data are expressed as means ± S.D. of three independent experiments. Single asterisks (*) indicate significant differences between CHO cells co-transfected with (filled bars) or without (open bars) gcHIF-1 in the absence of gcCITED, *p *< 0.05; double asterisks (**) indicate significant differences in luciferase activities of CHO cells co-transfected with pCMV-gcCITED or pCMV-gcCITEDΔCR2 in the presence of gcHIF-1, *p *< 0.05.

Previous work has demonstrated that the human CITED2 and CITED4 proteins are capable of competing with HIF-1 for specific binding to the CH1 domain of CBP/p300 and repressing the transcription of HIF-1 regulated genes [11,14]. To test whether gcCITED3a and gcCITED3b could also inhibit the transactivation activity of gcHIF-1, CHO cells were co-transfected with pBK-CMV-gcHIF1α and pBK-CMV-gcARNT2b, with or without pCMV-gcCITED3a or pCMV-gcCITED3b, together with the luciferase reporter construct pSV40-EpoHRE-Luc, which contains four copies of the HRE from the human *EPO *gene [25]. As shown in Figure 6, the gcHIF-1-mediated transactivation of HRE-luciferase activity in CHO cells was reduced significantly (ca. -40%) in the presence of gcCITED3a or gcCITED3b (*p *< 0.05). The CR2 domains of CITED2 and CITED4 have been shown to be involved in the inhibition of HIF-1 transcriptional activity in humans [11,26]. To determine whether the CR2 domain of gcCITED3a/gcCITED3b is required for the inhibition of gcHIF-1 activity, CHO cells were transfected with pBK-CMV-gcHIF1α and pBK-CMV-gcARNT2b, together with pCMV-gcCITED3aΔCR2 or pCMV-gcCITED3bΔCR2 (deletion mutants lacking the CR2 domain) expression vectors. No significant reduction in gcHIF-1 transactivation of HRE-luciferase activity was observed in the presence of either of these two CR2-deletion derivatives (Figure 6), indicating that the negative regulatory effect of gcCITED3a and gcCITED3b on gcHIF-1 is dependent upon the CR2 domain of these proteins. Next, to determine whether the inhibition of the gcHIF-1-mediated transactivation of HRE-luciferase activity may be due to reduced synthesis of gcHIF-1α caused by ectopic expression of gcCITED3a or gcCITED3b, Western blot analysis was performed using an anti-gcHIF-1α antibody [23]. CHO cells transfected with pBK-CMV-gcHIF-1α alone or co-transfected with pCMV-gcCITED3a or pCMV-gcCITED3b contained comparable levels of the gcHIF-1α protein (data not shown), which indicated that the decrease in gcHIF-1 transcriptional activity in the presence of gcCITED3a/gcCITED3b is not due to reduced gcHIF-1 synthesis.

### gcCITED3a and gcCITED3b physically interact with CBP/p300

**Figure 7:**
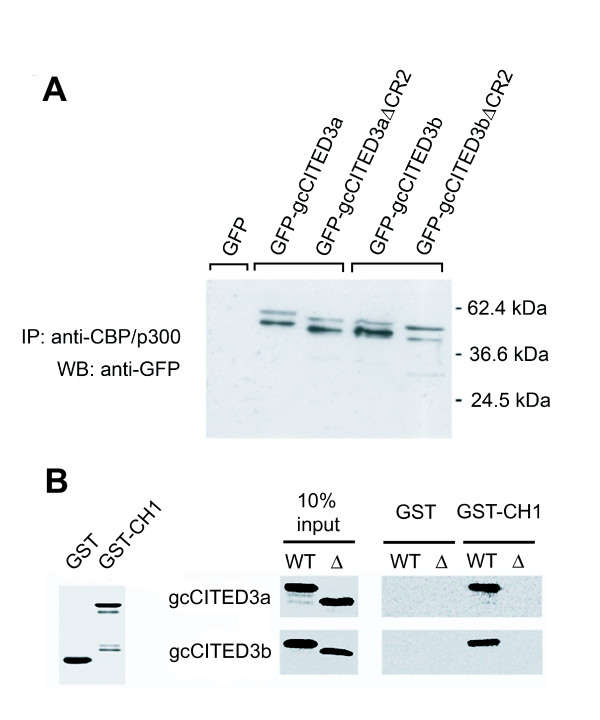
**gcCITED3a and gcCITED3b physically interact with CBP/p300**. *A*. Immunoprecipitation and Western blot analysis. CHO cells were separately transfected with pGFP-gcCITED3a, pGFP-gcCITED3aΔCR2, pGFP-CITED3b, or pGFP-CITED3bΔCR2, and whole cell lysates prepared and immunoprecipitated with anti-CBP antibody (Santa Cruz). The immunoprecipitates were resolved on 12% denaturing SDS-PAGE and Western blot analysis was carried out to detect GFP fusion proteins using anti-GFP antibody (Santa Cruz). *B*. GST pull-down assays. GST protein only or GST protein fused to gc-p300/CH1 (CH1 domain of grass carp p300) was purified from bacterial culture and immobilized on glutathione-Sepharose beads. *In-vitro*-transcribed and -translated [^35^S]-methionine-labeled gcCITED3 or gcCITED3ΔCR2 was incubated with purified GST-fused CH1 or GST alone as indicated in the figure.

Since gcCITED3a and gcCITED3b have been shown to repress the transcriptional activity of gcHIF-1 (presumably via competition for the CBP/p300 co-transactivator), we used immunoprecipitation and GST pull-down assays to determine whether these proteins can directly interact with CBP/p300. CHO cells were transfected with pGFP-gcCITED3a, pGFP-CITED3b, pGFP-gcCITED3aΔCR2 or pGFP-CITED3bΔCR2, and whole cell lysates were prepared and immunoprecipitated with an anti-CBP antibody (Santa Cruz). Western blot analysis of the anti-CBP immunoprecipitates using an anti-GFP antibody (Santa Cruz) (Figure 7A) demonstrated that both gcCITED3a and gcCITED3b co-immunoprecipitate with the endogenous CBP/p300 proteins in the CHO cells. This result suggests that both proteins are capable of physically interacting with CBP/p300. Since the interaction of human CITED2 and CITED4 with CBP/p300 has been shown to be dependent on the CR2 domain [11,26], we deleted this domain in gcCITED3a and gcCITED3b. Interestingly, co-immunoprecipitation was also observed between the mutant GFP-gcCITED3aΔCR2 and GFP-gcCITED3bΔCR2 proteins and CBP/p300, while GFP alone did not show any interaction. Taken together, these results indicate that, unlike hCITED2 and hCITED4, the CR2 domain in gcCITED3a and gcCITED3b is not required for physical interaction of the proteins with CBP/p300.

Using GST-pull down assays, we next determined whether gcCITED3a and gcCITED3b physically bind to the CH1 domain of the grass carp p300 protein. The CH1 domain of grass carp p300 (gc-p300/CH1) [GenBank: EU450671] was amplified using degenerate primers and subcloned into the pGEX-5X-2 vector (Promega). GST and GST-gc-p300/CH1 fusion proteins were incubated with *in-vitro*-transcribed and -translated [^35^S] methionine-labeled gcCITED proteins, followed by fractionation on SDS-PAGE. As shown in Figure 7B, only the wild-type gcCITED3a and gcCITED3b proteins, but not the gcCITED3aΔCR2 or gcCITED3bΔCR2 deletion derivatives, were pulled down by the GST-gc-p300/CH1 fusion protein, demonstrating that the CR2 domain facilitates specific binding of both gcCITED3a and gcCITED3b to the CH1 domain of CBP/p300.

## Discussion

CITED3 is the least studied member of the CITED family, and the molecular targets and specific mechanisms underlying the action of CITED3 have not yet been clearly identified. In this study, we have characterized two closely-related CITED3 genes - *gcCited3a *and *gcCited3b *- in the hypoxia-tolerant grass carp, and *in silico *analyses demonstrated that these two CITED homologs are present only in the genomes of fish and are absent in mammals. Whilst we were able to recently identify CITED2 cDNAs from zebrafish and grass carp (unpublished observations), bioinformatic searches of all available fish genomes with various mammalian CITED4 proteins failed to turn up any CITED4-like sequences, which suggested that *Cited4 *genes are likely absent in fish. Interestingly, parsimony analysis of CITED proteins from diverse animal species has previously indicated that fish CITED3 proteins are most closely related to the mammalian CITED4 [20]. This raises the possibility that CITED3 and CITED4 are likely orthologous proteins that may have arisen subsequent to lineage divergence between mammalian and non-mammalian vertebrates, and that CITED3a and CITED3b have descended from a gene duplication event in fish from an ancestral CITED3 gene.

We have shown that the *gcCited3a *and *gcCited3b *genes are transcriptionally up-regulated by the gcHIF-1 transcription factor (Figure 4), and the gene products, in turn, repress gcHIF-1 transcriptional activity at the molecular level (Figure 6). As *gcCited3a *and *gcCited3b *are differentially activated transcriptionally by hypoxia in certain fish tissues (Figure 3), our results suggest that gcCITED3a/gcCITED3b may serve to auto-regulate the HIF transcriptional response in hypoxic cells of the corresponding tissues. Interestingly, gene transfection studies demonstrated that gcHIF-1 transcriptional activity was inhibited by ectopic expression of the wild-type gcCITED3a/gcCITED3b, but not the mutant gcCITED3aΔCR2 or gcCITED3bΔCR2 proteins, which provided compelling evidence that the observed inhibition of HIF-1 activity is mediated by the CR2 domains of gcCITED3a and gcCITED3b. While GST pull-down assays demonstrated that the CR2 domains of gcCITED3a and gcCITED3b are indeed required for specific binding of the proteins to the CH1 domain of the CBP/p300 co-transactivator (Figure 7B), co-immunoprecipitation experiments showed that gcCITED3a, gcCITED3b, and the corresponding ΔCR2 mutant proteins could all physically interact with the endogenous CBP/p300 in CHO cells (Figure 7A). These results are consistent with the notion that both gcCITED3a and gcCITED3b can physically interact with CBP/p300 at multiple sites apart from the respective CR2 and CH1 domains of these proteins, and the functional implications of which await further investigations. To our knowledge, this is the first study demonstrating physical interaction of CITED3 proteins with p300, and that CITED3 can negatively regulate HIF-1 transcriptional activity in a manner similar to the mammalian CITED2 and CITED4 proteins [11,27], which require their CR2 domains to exert this function.

*In vivo *regulation of the *gcCited3a *and *gcCited3b *genes by HIF-1 in the kidney and liver tissues of hypoxic fish was confirmed by ChIP-PCR assays, which revealed that endogenous gcHIF-1α is recruited to the *gcCited3a *promoter at region 4 (which contains a single core HRE at -59/-56) (Figure 5A). In contrast, three different ChIP patterns were observed with the *gcCited3b *promoter. Most notably, gcHIF-1 binding was detected in *gcCited3b *in the kidney of hypoxic fish (but not normoxic kidney) at regions 1 (nt positions -1731/-1136) and 2 (nt positions -1257/-742); neither of which contains any consensus HRE sequence (Figure 5B). These observations are not surprising as it has been previously shown that HIF-1 is expressed in many organs in mammals [28] and fish [29], albeit at varying levels, under both normoxic and hypoxic conditions. What was surprising, however, was the binding of gcHIF-1 to two HRE-less regions in *gcCited3b*. A closer inspection of the ChIP-positive regions of the *gcCITED3a *and *gcCITED3b *promoters also failed to identify any sequence motifs that resemble the non-canonical HRE sequence (GATGTG) recently reported in killifish [30]. Indeed, the binding of HIFs to gene promoters that lack the consensus HRE motif (NRCGTG) have been reported recently for human HIF-1 and HIF-2 using ChIP [31], whereby the binding of transcription factors to promoter regions lacking the consensus recognition sequence may be attributed to the formation of higher order DNA/protein complexes [31-33].

Experiments are currently underway in our lab to further explore the selectivity of HIF-1 binding to DNA that lacks the consensus HRE motif, 5'-RCGTG-3'. Notwithstanding, our findings in this study indicate that the *gcCited3a *and *gcCited3b *genes interact differentially under normoxia and hypoxia with gcHIF-1 in kidney and liver tissues, presumably via different sets of distal enhancer binding proteins to achieve differential gene expression.

Northern blot analysis revealed that both *gcCited3a *and *gcCited3b *are ubiquitously expressed in all fish tissues examined, except muscle (Figure 3). Although they share a similar overall mRNA expression pattern, the predominant expression sites of *gcCited3a *and *gcCited3b *are the kidney and liver, respectively. This raises the question of whether these two genes have independent or partially redundant functions in these tissues. The major structural difference of these two homologs with other CITED subtypes is the presence of a QH-rich domain in both gcCITED3a and gcCITED3b, which may contribute to functional differences. In addition to their known involvement in the regulation of HIF-1 signaling, a growing body of evidence indicates that the CITED proteins can interact with a variety of signaling molecules, suggesting that these proteins may have a role in diverse cellular processes. For example, while CITED1 is able to activate Smad4-dependent transcription, it has also been shown to repress the Wnt/β-catenin signaling pathway, which regulates early nephronic patterning in vertebrates [34]. CITED2, on the other hand, interacts with Smad2 and Smad3 to induce expression of the matrix metalloproteinase 9 gene in TGF-β-mediated tumor cell invasion [16]. In addition, CITED2 and CITED4, along with some isoforms of the transcription factor AP2, have been shown to activate genes that may be involved in neural crest, neural tube, and cardiac development [14,35]. Moreover, CITED1 and CITED2 were shown to interact with ER-α and PPAR-α, respectively, to activate the transcription of target genes [7,15]. Overall, based on the phenotypes of their gene knockouts and their interactions with many different and important developmental signaling pathways, it appears that members of the CITED family are versatile transcriptional cofactors or effector molecules. These genes likely engage in diverse cellular and developmental processes/functions and serve to fine-tune a number of signaling pathways to mediate their ultimate biological functions.

All known interactions between CITED proteins and various other transcription factors are bridged by the common transcriptional cofactor CBP/p300. This cofactor is required to mediate either the transcriptional gene activation or repression mechanisms of the CITED proteins. During gene activation, CITED proteins interact with CBP/p300 via the CR2 domain to form a transcription complex along with other transcription factors, such as Smad3, Smad4, or AP2A, B, or C [14,16,35-37]. This complex induces the transcription of specific target genes. Conversely, during gene repression, CITED proteins may compete with specific transcription factors, such as HIF-1α or β-catenin [11,26,34], for a common binding site on the CBP/p300 protein. This binding diminishes the transactivation activity of the latter transcription factors. Based on the studies of mammalian CITED genes, with which the grass carp CITED proteins share significant sequence homology (in particular the CR2 domain), and the ability to bind CBP/p300, it is likely that the gcCITED proteins may interact with the equivalent transcription factors described in mammalian systems to regulate similar cellular processes in fish.

Both the *gcCited3a *and *gcCited3b *genes can be transactivated by subjecting fish to both short- and long-term exposure to low oxygen tension. Long-term exposure led to a dramatic over-expression of both *gcCited3a *and *gcCited3b *in specific tissues, suggesting that certain processes in these tissues require gcCITED3a or gcCITED3b or that the CITED3a or CITED3b proteins directly alleviate the effect of hypoxia. The gene responses to hypoxia by other members of the CITED family, and their roles in development, indicate that these proteins may be responsible for controlling gene transcription during tissue development, which may be driven by slight hypoxia in the developing tissues [38].

## Conclusion

Taken together, *gcCITED3a *and *gcCITED3b *genes are differentially expressed and regulated in different fish organs in response to hypoxic stress. Both genes are activated by gcHIF-1 in gene transfection studies, and ChIP assays comparing normal and hypoxic conditions reveal differential in vivo binding of gcHIF-1 to both gene promoters in kidney and liver tissues. HRE-luciferase reporter assays showed that both gcCITED3a and gcCITED3b proteins inhibit gcHIF-1 transcriptional activity, presumably by binding specifically to the CH1 domain of the grass carp p300 protein as confirmed by GST pull-down assays.

## Methods

### Fish Culture and Treatment

Animal care and experimentation were undertaken in accordance with City University of Hong Kong animal care guidelines. Grass carp (*Ctenopharyngodon idella*) of ca. 500 g body weight were obtained from a fish hatchery in Panyu, Guangdong, China and maintained in 300-l fiberglass tanks with circulating, filtered and well-aerated water at 20 ± 1°C under a 12:12 h day:night cycle. Fishes were reared under normoxia (7.0 ± 0.2 mg O_2_/l) or hypoxia (0.5 ± 0.3 mg O_2_/l) for 4 and 96 h in a continuous flow system as previously described [39]. Dissolved oxygen (DO) was monitored continuously using a YSI Model 580 dissolved oxygen meter (Geo Scientific Inc., Canada). After the exposure period, the fishes were anaesthetized and tissues were immediately dissected out, snap-frozen in liquid nitrogen and stored at -80°C until ready to be processed.

### RNA Isolation and Cloning of Full-length cDNAs

Total RNA was isolated using the TRIZOL reagent (Invitrogen, USA) according to the manufacturer's instructions. Poly(A)^+ ^RNA was isolated from total RNA using the PolyATtract mRNA Isolation System III kit (Promega, USA) according to the manufacturer's instructions. Cloning of the full-length gcCITED3a cDNA sequence has been previously reported by our group [20]. To facilitate cloning of the full-length gcCITED3b cDNA, 5'- and 3'-RACE PCR were performed using the Marathon Amplification Kit (Clontech, USA). Briefly, first-strand cDNA was synthesized from 1 μg kidney poly(A)^+ ^RNA of hypoxic fish using the cDNA Synthesis Primer and AMV reverse transcriptase. Second-strand cDNA was generated in an enzymatic reaction containing DNA polymerase I, DNA ligase, and RNase H from *E*.*coli*. Double-stranded cDNA was blunt-ended with T4 DNA polymerase and ligated to Marathon cDNA Adaptor. For 5'-RACE, adaptor-ligated ds-cDNA was used as the template in PCR amplification using adaptor primer AP1 and antisense gene-specific primer 5' GSP1 (5'-CCACCACTTATCTGGCCGTTGACCT-3'), derived from the partial gcCITED3b cDNA sequence. Nested PCR was performed using nested adaptor primer AP2, and gene-specific primer 5' GSP2 (5'-ACGCCATTCATCACGTTCCTCTGCC-3'). For 3'-RACE, PCR amplification of the ds-cDNA was performed with primer AP1 and gene-specific primer 3' GSP1 (5'-CCTTACAGCATCTCAGCAGCTTATGGC-3'). Nested PCR was performed with AP2 and gene-specific primer 3' GSP2 (5'-CACAGAATGGGTCCTGCTCAGTTGG-3'). The full-length gcCITED3b cDNA was cloned by RT-PCR using forward and reverse primers targeting the 5'- and 3'-RACE products, respectively, with *Pfu *DNA polymerase (Promega). The PCR profile consisted of: denaturation at 95°C for 2 min followed by 30 cycles of 95°C for 1 min, 60°C for 30 s and 73°C for 3 min; and a final extension step at 73°C for 5 min. The full-length cDNA was confirmed by DNA sequencing.

### Preparation of Reporter Constructs and Expression Vectors

The 5'-flanking sequences of *gcCited3a and gcCited3b *were obtained using the GenomeWalker Universal Kit (Clontech) according to the manufacturer's instructions. Briefly, 2.5 μg grass carp genomic DNA was digested separately with the restriction enzymes *Dra*I, *Eco*RV, *Pvu*II or *Stu*I. GenomeWalker Adaptor was ligated to the purified DNA and ligation products were subjected to two rounds of PCR (primary and secondary PCR) using the Advantage Genomic Polymerase Mix (Clontech). The touch-down PCR profiles for primary and secondary PCR consisted of denaturation at 94°C for 2 min followed by 5 cycles of denaturation at 94°C for 30 s and annealing at 68°C for 30 s, and 25 cycles of annealing at 65°C for 30 s and extension at 68°C for 3 min. This is followed by a final extension at 68°C for 5 min. Primers CITED3a-F (5'-GTGTCTCAATCTAGTGAGCTGCCTACC-3') or CITED3b-F (5'-CAGTTGAAGCTCTGCGTAGCAATACTGTTC-3') and AP1 were used in primary PCR, while primers CITED3a-R (5'-CGGCAGGACAATTGAGCTTTATCTGTTC-3') or CITED3b-R (5'-GGTCCAGCCTTCTCGTTATCACAGCT-3') and AP2 were used in the secondary PCR. Amplification products were purified and cloned into plasmid vectors for DNA sequencing. The gcCITED3a (1817 bp) and gcCITED3b (1713 bp) constructs used in this study were cloned into the promoterless pGL3-Basic luciferase reporter plasmid (Promega). Vectors expressing the grass carp HIF-1α (pBK-CMV-gcHIF-1α) and ARNT2b (pBK-CMV-gcARNT2b) were constructed as previously described [23]. Coding sequences for gcCITED3a, gcCITED3b, the gcCITED3aΔCR2 and gcCITED3bΔCR2 deletion derivatives were amplified by PCR and subcloned into the pCMV-TNT expression vector (Promega) using standard procedures. The coding sequence of the CH1 domain of the grass carp p300 protein was PCR-amplified using gene-specific primers gcp3GST-F (5'-GAGTATCGAATTCCTCAGCATGGGCAG-3'; *Xho*I site is underlined) and gcp3GST-R (5'-TATCTCGAGTCACAGCATTCCCGGATG-3'; *Eco*RI site is underlined), and the PCR product was subcloned into the *Xho*I/*Eco*RI sites of the pGEX-5X-2 GST vector (GE Healthcare, USA) to yield pGST-gcCH1. GFP-tagged gcCITED3a, gcCITED3b, gcCITED3aΔCR2 and gcCITED3bΔCR2 proteins were produced by subcloning the ORF of the corresponding cDNAs into the pCMV-GFP expression vector (Stratagene, USA).

### Northern Hybridization Analysis

Total RNA (13.5 μg) was electrophoresed on 1% (w/v) agarose/formaldehyde gel in 1× MOPS buffer (20 mM MOPS, pH 7.0, 5 mM sodium acetate, 1 mM EDTA) and blotted onto Hybond-XL membrane (GE Healthcare). Blots were prehybridized at 65°C for 30 min in ExpressHyb solution (Clontech) and Northern hybridization carried out at 65°C for 2 h in the same solution containing 2.0 × 10^6 ^cpm/ml of [^32^P]dCTP-labeled cDNA probe prepared by random priming (GE Healthcare). Blots were washed thrice in 2× standard sodium citrate (SSC), 0.05% (w/v) sodium dodecyl sulphate (SDS) for 10 min at room temperature, and twice in 0.1× SSC, 0.1% SDS (w/v) for 20 min at 50°C. Blots were exposed on a phosphor screen (Kodak, USA) at room temperature for 20 h, and the signals were captured and quantified using the Molecular Imager FX System (Bio-Rad, USA). A 115-bp 28S rDNA fragment (and confirmed by DNA sequencing) was amplified from grass carp total DNA using primers 28S-F (5'-GATCCTTCGATGTCGGCTCT-3') and 28S-R (5'-CTAACCTGTCTCACGACGGT-3') and used as an internal control probe in Northern hybridization for normalization of gene expression.

### Transient Transfection and Luciferase Reporter Assays

CHO cells (kind gift from Professor Peter Ratcliffe of the Wellcome Trust Center for Human Genetics, Oxford University, UK) were seeded in 24-well plates at 1 × 10^5 ^cells per well and transfected with 120 ng of p(HRE)_4_-Luc reporter plasmid and 60 ng of pSVβ-Gal expression vector (Promega) using LipofectAMINE 2000^® ^transfection reagent (Invitrogen) in OptiMEM^® ^I reduced serum medium according to the manufacturer's recommendations. The p(HRE)_4_-Luc reporter plasmid consists of 4 copies of the human erythropoietin hypoxia-responsive element (HRE) linked to the SV40 promoter and firefly luciferase gene (a gift from Professor Yoshiaki Fujii-Kuriyama, the Center for Tsukuba Advanced Research Alliance and the Institute of Basic Medical Sciences, University of Tsukuba, Japan). Ectopic expression of gcHIFα involved cotransfection of 120 ng of pBK-CMV-gcHIF-1α or an equimolar amount of the empty pBK-CMV vector with the luciferase and β-Gal reporter plasmids in the presence of 120 ng of pBK-CMV-gcARNT2b. The total DNA used in each transfection was adjusted to 1 μg by adding appropriate amount of pcDNA3 vector. Approximately 24 h post-transfection, cells were harvested and luciferase activity was measured using the Bright-Glo™ Luciferase assay kit (Promega) and normalized to β-galactosidase activity to correct for variations in transfection efficiency. The relative luciferase activity of the pGL3-Basic vector was set at 1.0 RLU for each data set. Data are expressed as means ± S.D. from three independent experiments with three replicates per sample in each experiment.

### Co-immunoprecipitation Assay

CHO cells (1 × 10^6^) were transfected with 10 μg of pCMV-GFP or pCMV-GFP-gcCITED3a, pCMV-GFP-gcCITED3b, pCMV-GFP-gcCITED3aΔCR2 or pCMV-GFP-gcCITED3bΔCR2. Twenty hours post-transfection, the cells were washed in ice-cold 1× PBS buffer and lysed in 0.5 ml TNTE buffer at 4°C. Cell lysate was incubated overnight at 4°C with mouse anti-CBP/p300 antibody (Santa Cruz, USA) and then incubated with Protein A/G agarose (Santa Cruz) at 4°C for 1 hour. Unbound proteins were removed by washing with TNTE buffer and bound proteins were denatured by boiling in SDS-PAGE buffer followed by Western blot analysis using anti-GFP antibody (diluted 1:5000, B-2, Santa Cruz).

### Western Blot Analysis

GFP-fusion proteins were separated in 12% SDS-PAGE, transferred to nitrocellulose membrane (Bio-Rad), blocked in 4% non-fat dry milk in 0.1% TPBS (0.1% Tween 20 in 0.1 M Na_2_HPO_4_, 0.1 M NaH_2_PO_4_, 0.5 M NaCl pH 7.2) for 30 min, and the membrane was incubated overnight at 4°C with anti-GFP antibody B-2 (diluted 1:5000; Santa Cruz) in the blocking medium. Non-specific binding of antibody was washed off with three changes of 0.1% TPBS followed by detection with 1:5000 diluted HRP-conjugated secondary anti-mouse IgG (Zymed, USA) at room temperature for 1 h using the ECL Plus Detection System (GE Healthcare). Western blot detection of gcHIF-1α and β-tubulin proteins was carried out as previously described [23].

### Glutathione S-transferase (GST)-pull-down Assay

GST-fusion proteins (or GST alone) were induced in *E. coli *DH5α for 30 min in 10 mM isopropyl-β-D-thiogalactopyranoside (IPTG). Cells were lysed and GST-fusion proteins were prebound with 30 μl of glutathione-Sepharose beads. The beads were incubated with the *in-vitro*-transcribed and -translated [^35^S] methionine-labeled proteins for 3 - 4 h at 4°C. The beads were washed three times with washing buffer, and analyzed in 15% SDS-PAGE, and protein bands were visualized using the FX phosphoimaging system (Bio-Rad).

### In Vitro Translation

The pCMV-gcCITED3a, pCMV-gcCITED3b, pCMV-gcCITED3aΔCR2 and pCMV-gcCITED3bΔCR2 plasmids were transcribed *in vitro *and translated using the TNT T7 Quick coupled transcription/translation system (Promega) in the presence of [^35^S] methionine according to the manufacturer's instructions. The translated proteins were analyzed in 15% SDS-PAGE and images visualized using the FX Phosphoimaging system (Bio-Rad).

### Chromatin Immunoprecipitation (ChIP) Assay

Five separate normoxia/hypoxia exposure experiments were carried out, and for each, grass carp (ca. 2.5 kg each) were acclimatized for one week in normoxic water and then exposed to hypoxia (n = 2) or normoxia (n = 2) for 4 h. Chromatin immunoprecipitation (ChIP) assay was performed on kidney and liver tissues from these fish according to the instructions of the ChIP assay kit (Upstate, USA). Kidney (0.8 g) and liver (1.6 g) tissues were fixed in 1% formaldehyde (Sigma-Aldrich, USA) at room temperature for 15 min, and the cross-linking reaction was terminated with 125 mM glycine (Sigma-Aldrich) for 5 min. Tissues were homogenized using a Dounce homogenizer in ice-cold PBS containing 1× protease inhibitor cocktail (Sigma-Aldrich) and then sonicated six times for 15 s each in lysis buffer (1% SDS, 10 mM EDTA, 50 mM Tris-Cl, pH 8.0, 1× protease inhibitor cocktail, and 1 mM PMSF) at 55% amplitude using a Cole Palmer Ultrasonic Processor to generate DNA fragments of 500 - 800 bp. For immunoprecipitation, a rabbit anti-gcHIF-1α polyclonal antibody AB-4 [23] was used. Immunoprecipitated DNA was amplified by PCR using primers spaced 200 - 400 bps apart and encompassing regions that contain putative hypoxia-responsive elements (or HREs) within the *gcCited3a *and *gcCited3b *gene promoters (Figure 3). For the positive control (input), purified chromatin without the immunoprecipitation step was used for PCR. The PCR profile consisted of 35 cycles of denaturation at 94°C for 30 s, annealing at 55°C for 45 s, followed by extension at 72°C for 1.5 min. PCR was performed in a 25-μl reaction mixture containing 5 μl of purified DNA, 0.2 mM dNTP, 0.2 μM each of forward and reverse primer, 1× PCR buffer and 0.3 U of *Taq *DNA polymerase.

### Phylogenetic Analysis

Phylogenetic analysis was performed by maximum parsimony using the Molecular Evolutionary Genetics Analysis (MEGA) Version 3.1 program http://www.megasoftware.net/. Evaluation of the inferred phylogeny was performed using the bootstrap test with 1000 replications.

### Statistical Analysis

A non-parametric χ^2 ^test was used to test the null hypothesis that the ratio of hypoxic:normoxic mRNA expression level was not significantly different from 1 [40]. One-way ANOVA was used to examine inhibitory effects of ectopic expression of gcCITED3a and gcCITED3b on gcHIF-1α induction on HRE-driven luciferase activity. Where significant effects were detected, Tukey's tests were performed to identify significant difference between individual means; α = 0.05 was used in all statistical tests.

## List of abbreviations

gcCITED: grass carp cAMP-responsive element-binding protein (CBP)/p300-interacting transactivator with glutamic acid/aspartic acid-rich tail; ChIP: chromatin immunoprecipitation; HIF: hypoxia inducible factor; HRE: hypoxia-responsive element; GST: glutathione-S-transferase; CH1: cysteine/histidine-rich domain; CBP: CREB-binding protein.

## Authors' contributions

PKSN designed and carried out most of the experimental work described in this paper. TFNK performed the ChIP experiments, RMKY assisted with many of the expression studies, MMLW contributed to the sequence analysis. SKC and RYCK contributed to the design and planning of this study, and writing of the manuscript. All authors read and approved the final manuscript.
